# Clinical outcomes of twice-weekly teriparatide acetate administration in osteoporosis

**DOI:** 10.1007/s11657-025-01622-4

**Published:** 2025-11-19

**Authors:** Ayako Tominaga, Hideyuki Maruki, Keiji Wada, Yasushi Terayama, Hideharu Nishi, Yoshiharu Kato, Ken Okazaki

**Affiliations:** 1 Department of Orthopedic Surgery, Tokyo Women’s Medical University, Tokyo, Japan; 2 Department of Orthopaedic Surgery, Saitama Medical University, Saitama, Japan; 3 Shiseikai Daini Hospital, Tokyo, Japan; 4 Spine Center, Tomei Atsugi Hospital, Atsugi, Kanagawa Japan; 5 Hasuda Hospital, Saitama, Japan; 6 Kita Shinagawa 3rd Hospital, Tokyo, Japan

**Keywords:** Bone mineral density, Continuation rate, Osteoporosis, Side effects, Teriparatide acetate

## Abstract

***Summary*:**

W2TPD, a twice-weekly teriparatide administration regimen, was used on 163 patients. The continuation rate was 47%, with only one new fracture. Even after performing antiresorptive therapy, spine BMD increased significantly in the majority of groups. W2TPD demonstrated good efficacy and tolerability in a real-world sequential osteoporosis treatment model.

**Purpose:**

Teriparatide is the most commonly administered daily, but there are also once-weekly and twice-weekly regimens. The former demonstrated high efficacy in increasing bone mineral density (BMD) and preventing new fractures; however, the continuation rate was reported to be low due to a high incidence of side effects. As a result, the twice-weekly teriparatide administration schedule (W2TPD) was created. In this study, we conducted a real-world clinical evaluation of its efficacy as part of a sequential osteoporosis treatment regimen.

**Methods:**

The study included 163 patients with osteoporosis who were treated with W2TPD. Patients treated with W2TPD were divided into five groups based on their prior medication use: treatment-naïve (N), post-denosumab (post-D), post-bisphosphonate (post-B), post-romosozumab (post-R), and post-SERM (post-S). We examined treatment continuation rates, adverse events, and changes in BMD.

**Results:**

The overall treatment continuation rate was 47.9%, with only one patient developing a new fracture during treatment. Gastrointestinal side effects, such as heartburn, nausea, and vomiting, were common. The percent changes in spine BMD were 10%, 5.2%, 5%, − 1.5%, and 12.3% in the N, post-D, post-B, post-R, and post-S groups, respectively. Meanwhile, hips were found in 3.1%, 0.4%, 1.5%, 0%, and 2.2%, respectively. In terms of spine BMD, all groups except post-R had responder rates greater than 50%.

**Conclusion:**

The continuation rate of W2TPD was 47% and resulted in particularly favorable BMD gains in the spine. It was also discovered to be effective in increasing BMD even when following bisphosphonate treatment.

**Supplementary Information:**

The online version contains supplementary material available at 10.1007/s11657-025-01622-4.

## Introduction

Osteoporosis and fragility fractures are major health concerns in developed countries [1–4]. Fragility fractures have a significant negative impact on quality of life and life expectancy, with 1-yr mortality rates ranging from 6 to 28% for vertebral fractures and 15%–23% for proximal femoral fractures [5, 6]. Furthermore, many patients experience a decline in daily living activities, resulting in an unavoidable deterioration in quality of life. These issues are not limited to individual patients; they pose a national challenge. According to reports, healthcare costs for fragility fractures are rising globally, putting a financial strain on national economies around the world [4]. For these reasons, active treatment of osteoporosis is critical for preventing fractures.

Teriparatide acetate is a teriparatide formulation (TPD) developed by a Japanese company and first introduced in 2011. While the TPD developed overseas was for daily self-injection, this formulation was developed for once-weekly (W1TPD) administration in hospitals. Over 18 months, it demonstrated a favorable bone mineral density (BMD) increase of 6.4% in the lumbar spine and 3% in the hip [7, 8]. Notably, it had a significant effect on preventing new vertebral fractures, with a relative risk of 0.2 and an 80% fracture reduction rate [7]. However, previous studies frequently reported adverse effects, primarily gastrointestinal symptoms such as heartburn, nausea, and vomiting, resulting in a low continuation rate in real-world clinical practice [8–10]. To address this issue, a twice-weekly administration regimen of teriparatide acetate (W2TPD) was developed. W2TPD was developed as a self-injectable formulation, allowing patients to administer injections on any 2 days of the week [11]. The TWICE study, a clinical trial of W2TPD, found that while maintaining significant changes in bone turnover markers and BMD, this regimen also helped to reduce side effects and improve treatment adherence [8, 10, 11].

The W2TPD administration regimen has been shown to maintain the efficacy of W1TPD while decreasing the frequency of adverse effects; however, the literature on its real-world clinical outcomes is extremely limited [8, 10, 11]. Furthermore, no published studies have examined the changes in efficacy when W2TPD is used after previous treatment with other osteoporosis medications. The aim of our study was to provide concrete clinical evidence regarding the efficacy of W2TPD, particularly in terms of fracture incidence and its effect on BMD, as reports on W2TPD remain limited. Further, considering that treatment transitions are frequently required in real-world osteoporosis management, we also aimed to evaluate whether W2TPD retains its bone anabolic effects when used as a part of sequential therapy.

## Methods

In this study, we included all the patients with severe osteoporosis who began W2TPD treatment at Tokyo Women’s Medical University and its affiliated institutions between 2020 and 2023. W2TPD is a medication that is self-administered via subcutaneous injections at a dose of 28.2 μg twice a week, using an autoinjector (Teribone Auto Injector, Asahi Kasei Pharma Corp, Tokyo, Japan), in principle every 3 or 4 days (with 2 or 3 days between injection days). Treatment with the drug was administered to the patients, who visited our hospital or affiliated institutions, met the indication criteria for the drug, had no contraindications, and expressed a personal desire to receive the treatment. Severe osteoporosis was diagnosed when there were multiple fragility fractures or BMD of ≤  − 2.5 *SD* in the spine or hip [12]. The main reasons for switching medications were: (A) the occurrence of new fractures during treatment with other agents, indicating the necessity for treatment intensification; (B) the judgment that achieving a BMD above − 2.5 SD was unlikely to be attained with a continued use of the current medication; and (C) the development of medication-related osteonecrosis of the jaw, which made the continuation of antiresorptive therapy inappropriate [12]. The study excluded patients with open epiphyseal plates, bone metastases, or secondary hyperparathyroidism.

The primary endpoints were W2TPD administration regimen adherence rates and the number of new fractures. The secondary endpoints included adverse effects, changes in serum corrected calcium (Ca) levels, trends in bone turnover markers, changes in BMD, and responder rates. The analyzed medical records yielded information on continuation rates and adverse effects. Treatment discontinuation was performed either at a patient’s request or at a physician’s discretion when deemed necessary. Reasons for discontinuation were extracted from the patients’ medical records. Discontinuation times were divided into categories. Fracture occurrence was determined by patient-reported pain or trauma and confirmed by plain X-rays, with additional computed tomography or other imaging as required. Especially in vertebra fractures, plain radiographs were obtained in patients who reported new-onset back pain during regular follow-up visits. A vertebral fracture was defined as a reduction in vertebral height of ≥ 4 mm or ≥ 20% [13]. When radiographic assessment was inconclusive; however, clinical suspicion remained, additional MRI was performed. For asymptomatic vertebral fractures, we comparatively analyzed the plain radiographs taken before and after the initiation of teriparatide treatment. Blood samples were collected before starting W2TPD treatment and 1, 3, 6, 12, 18, and 24 months later. The corrected Ca values were calculated by measuring serum albumin and calcium levels. Procollagen type 1 N-terminal propeptide (P1NP) was identified as a bone formation marker, while tartrate-resistant acid phosphatase 5b (TRACP-5b) was designed as a bone resorption marker [14–17]. The P1NP was quantified using the Eclucis reagent (Roche Diagnostics, Minato-ku, Tokyo, Japan; Coefficient of Variation 3.0—5.0%) and enzyme-linked immunoassay (ECLIA). An Osteolinx kit (Nittobo Medical, Koriyama, Fukushima, Japan; Coefficient of Variation 2.1–5.6%) was used to measure TRACP-5b using an enzyme-linked immunosorbent assay. BMD was measured using dual-energy X-ray absorptiometry (DEXA) at the lumbar spine (L1–L4), total hip, and femoral neck. The DEXA equipment used in this study were Lunar iDXA (GE Healthcare, Chicago, USA) and Discovery (HOLOGIC, Marlborough, USA). Each patient underwent bone densitometry using the same device. We referred to the “mean T-score of healthy Japanese young adults aged 20 to 40 years” as the standard reference.

We used the least significant change (LSC) for BMD to assess the responder [18]. Based on the previous studies, we set the LSC for the spinal BMD change from the baseline to month 12 at 3% [18–22]. We used a 3% LSC change from baseline to assess efficacy at femoral neck and total hip, too [18, 23]. Those with a lower rate were classified as non-responders [18–22, 24]. Responders were evaluated separately for the spine, femoral neck, and total hip.

In contrast, it has been reported that osteoporosis medications can be influenced by previous treatments [23, 25–27]. As a result, we classified the patients based on the osteoporosis treatment they had received before starting W2TPD.

Groups were defined as follows:

- Patients who started osteoporosis treatment with W2TPD were classified as treatment-naïve (N).
- Patients switching from denosumab therapy were classified as post-DMAB (post-D) group.
- Patients who switched from bisphosphonate therapy were assigned to the post-BIS (post-B) group.
- Patients who switched from romosozumab therapy were classified as post-ROMO (post-R) group.
- Patients transitioning from selective estrogen receptor modulators (SERM) were assigned to the post-SERM (post-S) group.

Because both TPD and calcitriol formulations have the potential to raise serum calcium levels, they were rarely used together.

### Statistical analysis

Statistical analyses were performed using Easy R (EZR; Saitama Medical Center, Jichi Medical University, Saitama, Japan), an R interface [28]. Nonparametric methods were used for all analyses. The Kruskal–Wallis test was used to compare baseline patient characteristics across groups, followed by a post-hoc analysis using the Steel–Dwass test. The continuation rates were compared between groups using Fisher’s exact test. A *p* value of < 0.05 was considered statistically significant.

## Results

### Patient background

The total number of cases was 163. Table 1 reflects the patient’s background. The average age was 74.8 ± 9.5 yr, and 144 patients (87.8%) were female. The mean body mass index (BMI) was 22.0 ± 0.3. Two patients were being treated with eldecalcitol at the same time. Vitamin D2 or D3 preparations were not co-administered in any patients other than from these two cases. Calcium supplements were not used for any of the patients.

**Table 1:** Patient background

	*N* = 163
Sex: Female (%)	144 (87.8)
Age (yr)	74.8 ± 9.5
BMI (kg/m^2^)	22.0 ± 0.3
Mean eGFR (mL/min/1.73 m^2^)	64.4 ± 2.5
25 (OH) VitD (ng/mL)	17.8 ± 1.6
Factors in osteoporosis	
Primary osteoporosis (%)	122 (74.8)
Secondary osteoporosis (%)	41 (25.2)
History of fracture (*n*)	85
Vertebral	74
Proximal femur	13
Proximal humerus fracture	3
Distal radius	10
Atypical fracture	1
Spine T-score before starting W2TPD	− 2.2 ± 1.4
Total hip T-score before starting W2TPD	− 2.2 ± 0.9

*BMI* Body mass index; *eGFR*estimated Glomerular Filtration Rate, *TPD* Teriparatide

In terms of etiology, osteoporosis occurred in 122 cases (74.8%) as a result of primary osteoporosis, while 41 cases (25.2%) were classified as secondary osteoporosis. Supplement table 1 shows the breakdown of secondary osteoporosis in each group. Some patients had several concomitant conditions.

Eighty-five patients had concomitant fractures, with an average of 2.2 ± 0.2 vertebrae. Other fragility fractures were observed in 13 proximal femoral, 10 distal radius, three proximal humerus, and one atypical fracture. Some patients suffered multiple fractures. Before treatment, the average T-score was − 2.2 ± 1.4 (*SD*) for spine and − 2.2 ± 0.9 *SD* for the total hip.

Table 2 shows a comparative analysis of patient backgrounds across groups. The treatment groups included 98 patients in the N, 29 in the post-D, 16 in the post-B, six in the post-R, and 14 patients in the post-S groups. The treatment durations (in months) for each prior therapy were as follows: DMAB 24 [24–36], BIS 51 [28.5–64.5], ROMO 12 [8.2–12], and SERM 36 [24–42].

**Table 2:** Patient background by prior treatment

	*N*	Post-D	Post-B	Post-R	Post-S	*p* value
*N*	98	29	16	6	14	
Sex: Female (%)	82 (84.5)	28 (96.6)	14 (87.5)	5 (83.3)	14 (100)	0.264
Age	77 [70.0–82.0]	77 [73.0–79]	79.5 [76.8–82.3]	71.5 [52.0–72.3]	70 [67.0–73.5]	0.01
Secondary osteoporosis (%)	24 (24.5)	3 (10.3)	9 (56.2)	3 (50)	2 (14.3)	0.006
Correct Ca 0 M (mg/dL)	9.3 [9.1–9.6]	9.5 [9.4–9.9]	9.6 [9.5–9.75]	9.4 [9.1–9.7]	9.4 [9.2–9.5]	0.03
eGFR 0 M (mL/min/1.73 m^2^)	66.1 [50.5–77.0]	49.8 [45.0–59.4]	51.1 [39.5–63.0]	87.6 [80.8–91.4]	69.8 [64.5–72.1]	0.049
P1NP 0 M (µg/L)	53.0 [36.3–71.4]	14.6 [10.5–22.8]	27.6 [20.9–48.3]	67.7 [51.0–73.5]	31 [20.4–46.6]	< 0.001
TRACP-5b 0 M (mU/dL)	523.5 [312.3–803.8]	158.0 [120.3–211.3]	266.0 [188.0–363.0]	444 [167.0–459.0]	227 [155.5–303.5]	< 0.001
Spine T-score before starting W2TPD	− 2.6 [− 3.6 to − 1.8]	− 1.9 [− 2.4 to − 0.4]	− 2.1 [− 2.3 to –1.0]	− 2 [− 2.5 to − 1.6]	− 2.7 [− 3.3 to − 2.2]	0.108
Total hip T-score before starting W2TPD	− 2.3 [− 3.0 to − 1.7]	− 2.1 [− 2.6 to − 1.9]	− 2.5 [− 2.8 to –1.9]	− 2.1 [− 2.2 to − 2.1]	− 1.9 [− 2.4 to − 1.5]	0.537

*BMI* Body mass index; *Ca* Calcium; *eGFR*estimated Glomerular Filtration Rate, *P1NP* Procollagen Type1 N-Terminal Propeptide; *TRACP-5b* Tartrate-Resistant Acid Phosphatase 5b; *TPD* Teriparatide

The groups differed significantly in terms of age, secondary osteoporosis prevalence, renal function, P1NP, and TRACP-5b levels. The post-S group’s median age was significantly lower than the post-D and post-B groups (*p* = 0.029 and *p* = 0.026, respectively). Although the Kruskal–Wallis test showed a significant difference in renal function, a post hoc analysis revealed no clear intergroup difference. The post-D group showed significantly lower P1NP levels compared to the N, post-B, and post-S groups (*p* < 0.001, *p* = 0.015, and *p* = 0.01, respectively). The N group had significantly higher TRACP-5b levels than those of post-D, post-B, and post-S (*p* < 0.001, *p* = 0.042, and *p* = 0.009, respectively), while the post-B group had significantly higher values for TRACP-5b than the post-D (*p* = 0.025).

### Continuation rate and adverse events

Out of 163 cases, 78 patients were able to continue treatment, for a 47.9% success rate. The timing of treatment discontinuation was as follows: within 0–3 months in 72.9% of patients, 3–6 months in 1.2%, 6–12 months in 11.8%, and 12–24 months in 14.1%. When evaluating by treatment group, the final continuation rates were 44.9% in the N, 51.7% in the post-D, 56.3% in the post-B, 33.3% in the post-R, and 57.1% in the post-S groups, with no significant difference.

The reasons for treatment discontinuation included adverse events (*n* = 48), fear of self-injection (*n* = 5), insufficient efficacy (*n* = 3), cost (*n* = 3), poor compliance (*n* = 3), and unknown reasons (*n* = 29), with overlaps among categories. The reported adverse events (also with overlaps) included nausea, vomiting, or abdominal discomfort (*n* = 28); palpitations or hypotension (*n* = 9); headache or dizziness (*n* = 9); anxiety (*n* = 1); and chills (*n* = 1).

### Incidence of new fractures

Only one fracture occurred during W2TPD treatment, and the patient suffered a humeral fracture.

### Changes in blood test results

**Fig. 1:**
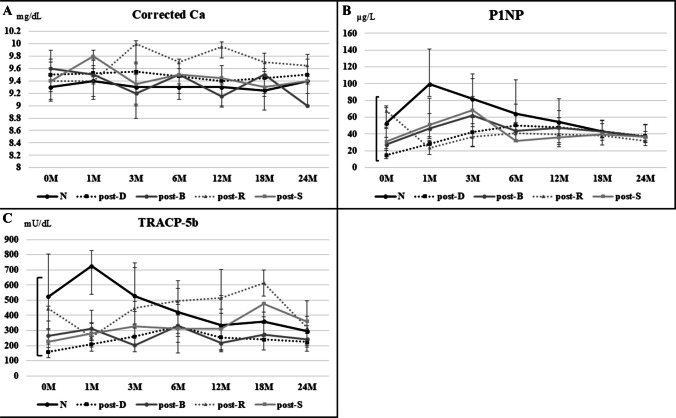
**A** Changes in corrected Ca levels by treatment group. During W2TPD treatment, no notable intergroup differences were observed in Ca dynamics. The figure shows the median values, alongside the range between the 25th and 75th percentiles. **B** Changes in P1NP levels by treatment group. While intergroup differences in P1NP levels were observed at the start of treatment, no significant differences were detected after 3 months. In all groups except for the post-R group, P1NP levels increased between 3 and 6 months and then gradually declined. In contrast, the post-R group exhibited a continuous decrease in P1NP levels even after W2TPD initiation. The figure shows the median values, alongside the range between the 25th and 75th percentiles. **C** Changes in TRACP-5b levels by treatment group. TRACP-5b levels varied between groups at baseline but showed no significant differences after 3 months of treatment. In the post-D and post-S groups, TRACP-5b levels gradually increased, whereas the post-R group exhibited a marked increase after 6 months. In contrast, the N and post-B groups showed stable or decreasing TRACP-5b levels over time. The figure shows the median values, alongside the range between the 25th and 75th percentiles

Figure 1A depicts the changes in corrected Ca levels across treatment groups. During the W2TPD treatment, there were no significant intergroup differences in Ca dynamics. Figure 1B depicts the changes in P1NP levels. While there were intergroup differences in P1NP at the beginning of treatment, the values in the groups did not differ significantly after 3 months. Except for the post-R, P1NP levels increased for 3 to 6 months before gradually decreasing. In contrast, the post-R group showed a consistent drop in P1NP levels even after the initiation of W2TPD.

Figure 1C depicts the changes in TRACP-5b levels, which showed significant differences between the groups at baseline but not after 3 months. In the post-D and post-S groups, TRACP-5b levels gradually increased, whereas in the post-R group, a significant increase occurred after 6 months. In the N and post-B groups, these levels remained stable or decreased over time.

### Bone mineral density changes

**Fig. 2:**
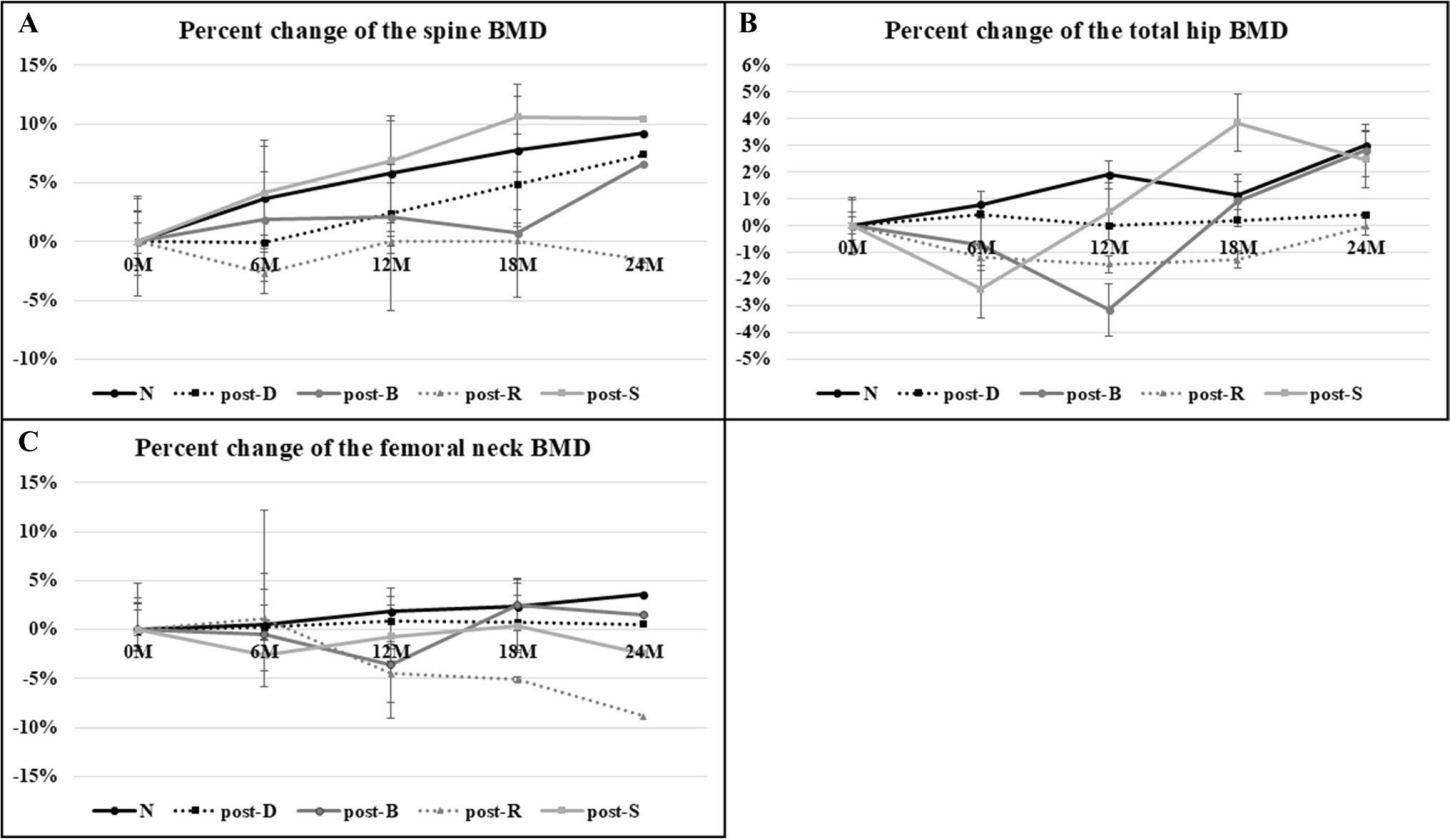
**A** Spine BMD changes by treatment group. All groups except for the post-R group showed a gradual increase in spine BMD over time. At the end of the 2-yr treatment period, the percentage changes in spine BMD were as follows: 10% in the N, 5.2% in the post-D, 5.0% in the post-B, − 1.5% in the post-R, and 12.3% in the post-S groups. No significant differences were observed between the groups. The figure shows the median values, alongside the range between the 25th and 75th percentiles. **B** Total hip BMD changes by treatment group. By the end of the 2-yr treatment period, the N, post-B, and post-S groups exhibited an increase in total hip BMD, while the post-D and post-R groups showed substantial changes. The percentage changes in total hip BMD were as follows: N group, 3.1%; post-D group, 0.4%; post-B group, 1.5%; post-R group, 0%; and post-S group, 2.2%. No significant intergroup differences were detected. The figure shows the median values, alongside the range between the 25th and 75th percentiles. **C** Femoral neck BMD changes by treatment group. The N and post-B groups demonstrated an increase in femoral neck BMD, whereas the post-D, post-R, and post-S groups exhibited either stability or a decline. By the end of the 2-yr period, the percentage changes in femoral neck BMD were as follows: N group, 3.1%; post-D group, − 1.8%; post-B group, 1.0%; post-R group, − 8.8%; and post-S group, 0.1%. The figure shows the median values, alongside the range between the 25th and 75th percentiles

Figure 2A–C shows the BMD change rates for the studied groups. All groups except the post-R showed a gradual increase in spine BMD over time. After the 2-yr treatment period, the spine BMD change rates were 10% in the N, 5.2% in the post-D, 5.0% in the post-B, − 1.5% in the post-R, and 12.3% in the post-S groups, with no significant differences.

In terms of total hip BMD, the N, post-B, and post-S groups all increased, whereas the post-D and post-R groups remained stable. By the end of the 2 yr, the total hip BMD change rates were 3.1% in the N, 0.4% in the post-D, 1.5% in the post-B, 0% in the post-R, and 2.2% in the post-S groups. Again, no significant differences were found between the groups.

In terms of femoral neck BMD, the N and post-B groups increased, whereas the post-D, post-R, and post-S groups remained stable or decreased. After 2 yr, the femoral neck BMD change rates were 3.1% in the N, − 1.8% in the post-D, 1.0% in the post-B, − 8.8% in the post-R, and 0.1% in the post-S groups.

A sex-based analysis of BMD changes was conducted using the Naive group as an example. While no significant differences were observed in the rate of changes in spinal or total hip BMD in femoral neck BMD was significantly higher in female patients (*p* = 0.064, *p* = 0.165, and *p* = 0.031).

### Responder rates

The responder rates by anatomical site are presented below. The responder rates for the spine were 90.5% in the N, 76.9% in the post-D, 66.7% in the post-B, 0% in the post-R, and 100% in the post-S groups. Except for the post-R, all groups had responder rates exceeding 50%.

For total hip, responder rates were 44.4% in the N, 30.8% in the post-D, 50.0% in the post-B, 0% in the post-R, and 20% in the post-S groups. Only the post-B group had a response rate of more than 50%.

For femoral neck, the responder rates were 55.6% in the N, 30.8% in the post-D, 50.0% in the post-B, 0% in the post-R, and 20% in the post-S groups. Only the N and post-B groups had response rates of more than 50%.

## Discussion

While daily administration is the cornerstone of a TPD treatment regimen, W1TPD and W2TPD formulations are also available. In this study, we evaluated the real-world clinical performance of the W2TPD administration regimen and confirmed its positive effects, such as increased treatment adherence, increased bone turnover markers, and improved BMD.

Notably, even in patients who had previously received antiresorptive medications, the W2TPD administration regimen resulted in a high responder rate in spine BMD. Our findings indicate that W2TPD can be effective even in patients who do not begin treatment with an anabolic-first approach, expanding the potential treatment strategies for osteoporosis in clinical practice.

TPD formulation is typically administered through a daily subcutaneous injection of 20 µg, but some regions in Asia use once-weekly (56.5 µg) or twice-weekly (28.2 µg) regimens. It has been reported that with daily TPD, bone formation increases first, followed by an increase in bone resorption, implying that bone formation is stimulated independently of bone resorption [29]. In contrast, with W1TPD, bone formation increases briefly and then decreases, and bone resorption is not activated [7]. Because of these differences in bone metabolism, the mechanisms of action and effects of daily and W1TPD administration regimens are thought to be distinct. With the former, a median treatment duration of 19 months resulted in a 9.7% increase in lumbar spine BMD and 2.8% in femoral neck [30]. Meanwhile, after 72 weeks of once-weekly administration, lumbar spine BMD increased by 6.4% and proximal femur BMD increased by 3% [7]. Daily and once-weekly teriparatide formulations have been reported to exert different effects on bone microarchitecture. While daily teriparatide increases cortical porosity, it also enhances bone strength by increasing cortical thickness [31–34]. In contrast, once-weekly teriparatide has been shown to increase both cortical thickness and area without inducing an increase in porosity. These effects have also been reported in the cortical bone of the proximal femur, which may explain the increase in proximal hip BMD observed in the present study. Furthermore, W1TPD has been shown to not only increase trabecular bone mass in lumbar vertebrae in an ovariectomized monkey model of postmenopausal osteoporosis but also to improve trabecular microarchitecture, improve bone quality parameters such as collagen content and collagen cross-linking, and, ultimately, increase bone strength [35].

While W1TPD is convenient and effective in maintaining high BMD, it has been linked to a high incidence of adverse events and a low continuation rate, estimated to be between 16.8% and 23.5% [8–10]. One proposed explanation for this low adherence is a relatively high dose administered at once due to the weekly dosing schedule, which results in the development of W2TPD [8, 10, 11]. In our real-world clinical study, the continuation rate for W2TPD was 47.9%. Compared with previously reported continuation rates for W1TPD, our findings were more than twice as high, indicating favorable outcomes. However, adverse effects—particularly gastrointestinal symptoms such as nausea, abdominal discomfort, and vomiting—were frequently reported. TPD has been shown to relax vascular smooth muscles, which may contribute to gastrointestinal symptoms [10]. To address these side effects, several strategies can be developed, including administering an injection before bedtime, staying hydrated, and taking concomitant medications [10, 36]. A study indicated that the incidence of headache with TPD was not different from that with placebo, whereas dizziness was reported to increase significantly with TPD [37]. Our adverse event reports included palpitations and dizziness. In this study, the dizziness observed was nonrotational and may have been related to factors such as blood pressure reduction [38–40]. The reported incidence of transient hypotension with TPD varies depending on the formulation used. A higher single dose of TPD is speculated to be associated with a higher incidence [38].

Because osteoporosis is a long-term condition, sequential therapy is frequently used [12, 26, 41–43]. Previous studies have found that the order in which osteoporosis medications are administered can have a significant impact on the efficacy of BMD improvement [12, 26, 41–43]. For example, switching from TPD to DMAB causes continuous BMD gains, whereas switching from DMAB to TPD may provoke either a transient or persistent decrease in BMD change [41, 44]. Switching from BIS to TPD can result in either a decrease or a smaller increase in BMD than treatment-naïve patients [26, 42]. Although the “anabolic-first” approach has recently received widespread support, it is not always feasible in real-world clinical practice [12, 45]. In the present study, W2TPD demonstrated a high responder rate for lumbar spine BMD, even when used after prior antiresorptive treatment. This suggests that W2TPD administration regimen may be a viable option in sequential therapy for cases where fractures occur during treatment with other osteoporosis drugs, in atypical fractures, or when a current medication is ineffective. However, switching from ROMO, a bone-forming agent, to W2TPD is best avoided. Although the post-ROMO group in our study was small, warranting further investigations, similar findings have previously been reported [46]. The underlying reason is unknown, but differences in the so-called anabolic window have been proposed as one possible explanation [46].

One of the main advantages of the W1TPD formulation is its high efficacy in preventing new fractures [7]. Similarly, in the present study, only one fracture occurred during W2TPD treatment, indicating a low rate of new fractures. More research is needed to confirm the fracture prevention effect of W2TPD, including comparative studies with other agents, a larger sample size, and a long-term follow-up.

Although the responder rates in the hip and femoral neck with W2TPD were less than 50%, previous studies have shown that the responder rate in the hip with ROMO is also < 50% [24, 47]. These findings suggest that the treatment effects of bone-forming agents alone may be unstable in terms of hip BMD. As a result, sequential therapy is even more important for effectively managing this site.

### Limitation

Some limitations must be addressed, including the small sample size, retrospective nature, and unequal number of participants of the same age and gender. This study, which recruited participants from Asia, may have had a bias. Smoking, a risk factor for osteoporosis, could not be evaluated in our study because it was not documented in the medical records. In this study, vitamin D and calcium supplements were not co-administered in most cases. However, in patients in whom hypercalcemia and hyperparathyroidism were excluded prior to treatment initiation, concomitant use of vitamin D may also be considered. We also did not check thyroid hormone levels. As this study has a retrospective observational design, the treatment continuation rate was reported as observed. The low continuation rate may have influenced the post-treatment outcomes. In particular, the number of patients, who transitioned from ROMO to W2TPD was very small, and the possibility of bias in the results cannot be excluded. Further studies with a larger sample size are necessary to validate these findings. Some reports have indicated that the use of TPD following DMAB may result in bone loss, and caution is warranted [41, 44]. In the present case series, no clinical fractures were observed in patients, who received W2TPD after DMAB; however, given the small sample size, it should be noted that the safety of this approach cannot be conclusively determined. Detailed assessments including asymptomatic fractures must be incorporated in future studies. We were unable to assess the efficacy of a combinative medication scheme due to its small size. Future research should be conducted with larger sample sizes and prospective designs.

## Conclusion

In this study, we conducted a real-world evaluation of the W2TPD over 2 yr and found favorable results, such as increased treatment adherence, increased bone turnover markers, and enhanced BMD. In the N group, the BMD increased by 10% in the lumbar spine, 3.1% in the total hip, and 4.9% in the femoral neck. Notably, even when W2TPD was combined with other osteoporosis medications, there was a high responder rate, particularly in spine BMD. These findings show that W2TPD can be effective even when an anabolic-first approach is not used, implying that this regimen expands the current therapeutic options for osteoporosis management.

## Supplementary Information

Below is the link to the electronic supplementary material.

Supplementary file1 (XLSX 16 KB)

## Data Availability

The data underlying this article will be shared upon reasonable request to the corresponding author.
